# A New Method for Erosion Prediction of 90° Elbow Based on Non-Axisymmetric Ultrasonic-Guided Wave and the PSO–LSSVM Algorithm

**DOI:** 10.3390/s23146311

**Published:** 2023-07-11

**Authors:** Zhaokun Wang, Sizhu Zhou, Ning Li, Yun Zeng, Gui Li

**Affiliations:** 1 School of Mechanical Engineering, Yangtze University, Jingzhou 434023, China; wangzhaokun950623@163.com (Z.W.);; 2 Hubei Key Laboratory of Mechanical Transmission and Manufacturing Engineering, Wuhan University of Science and Technology, Wuhan 430081, China; leegui2030@wust.edu.cn

**Keywords:** erosion monitoring, least squares support vector machine (LSSVM), fractional Fourier transform (FrFT), asymmetric ultrasonic-guided wave, particle swarm optimization (PSO)

## Abstract

The non-axisymmetric exciting guided wave can detect the thinning section of the elbow, and the time domain energy value of the signal collected at the outer arch position of the receiving end displays a downward trend as the remaining thickness of the erosion area decreases. To address the difficulty in detecting the erosion degree of the elbow with high accuracy, this paper uses the linear frequency modulation (LFM) signal to excite a non-axisymmetric guided wave that propagates in the 90° elbow and collects signals through four PZT receivers. To predict the erosion degree, the corresponding relationship between the energy value of the four signals after fractional Fourier filtering and the degree of elbow erosion is established through the particle swarm optimization (PSO)–least squares support vector machine (LSSVM) algorithm. The results show that the method proposed has an average accuracy rate of 98.1864%, 94.7167%, 99.119%, and 99.9593% for predicting the erosion degree of four elbow samples, and 94.0039%. and 81.2976% for two new erosion degrees, which are higher than the nonlinear regression model, LSSVM algorithm, and BP neural network algorithm. This study has guiding significance for real-time monitoring of elbow erosion.

## 1. Introduction

The pipeline system is a cost-effective way of transporting oil and gas, widely used in the petrochemical industry. As an essential part of changing the flow direction of the pipeline system, elbows are prone to erosion damage under the erosion of the internal conveying medium [1,2,3]. Therefore, it is necessary to accurately predict the remaining wall thickness.

Traditional nondestructive testing techniques have some limitations [4,5,6,7]. For example, magnetic particle testing can only detect ferromagnetic materials [8] and requires a low environment temperature; eddy current testing [9] can only detect conductive metal materials and non-metallic materials that can generate eddy currents; and radiographic testing [10] has high detection costs and slow detection speeds. Compared with these techniques, ultrasonic-guided wave detection has the advantages of small energy attenuation, an extensive detection range, and a fast detection speed. Therefore, it is more suitable for detecting the erosion degree of the elbow [11,12,13,14].

The pulse signal is generally used to excite axisymmetric guided waves to detect structural damage, and the position and depth of defects are judged by the amplitude and receiving time of echo signals. This method has a good detection effect on symmetrical parts [15,16]. However, when detecting the elbow, the following problems exist: the axisymmetric guided wave will produce non-axisymmetric reflection in the bending part of the elbow and mode conversion will occur, which increases the difficulty of identifying the corresponding mode from the echo signal; in some frequency ranges, it is difficult to ensure that a single axisymmetric mode is excited; axisymmetric guided waves are less effective in detecting axial and circumferential defects; when the test piece can only be contacted in a limited space, it is difficult to install sensor arrays required to excite axisymmetric guided waves [17,18]. Some scholars have tried to use non-axisymmetric guided waves to detect circular tube structures [19], cracks in rivet holes [20], and straight pipes [21,22] and have achieved good results. Therefore, we consider using non-axisymmetric guided waves to detect the erosion degree of elbow erosion.

The LFM signal has a large time-bandwidth product, a long detection distance, and a high-range resolution, widely used in radar, electronic communication, and fault diagnosis [23,24,25,26,27]. Its characteristics are suitable for detecting the erosion degree of the elbow. However, the strong coupling of the LFM signal in the time–frequency domain makes it difficult to separate the signal from the noise. Fractional Fourier transform (FrFT) has chirp basis decomposition characteristics, suitable for LFM signal filtering [28,29,30,31]. Some scholars have successfully detected cracks in reinforced concrete using the method of horizontal fusion of CODA waves and multi-ultrasonic sensor signals [32,33]. Inspired by this, we consider arranging multiple sensors on the elbow to receive signals and use machine learning algorithms to establish the internal relationship between the signals and the erosion degree.

Least squares support vector machine algorithm (LSSVM) has a fast solution speed and good robustness [34], and its characteristics are suitable for predicting the erosion degree of the elbow. The regularization parameter and kernel parameter affect the performance of LSSVM, and the determination of the values of these two parameters by empirical or grid methods suffers from subjectivity and arbitrariness, making the results inaccurate. Particle swarm optimization (PSO) is an evolutionary algorithm with fast convergence speed, a simple solution process, and global search capability, which can effectively optimize the above two parameters [35,36].

Based on the above discussion, to solve the problem that it is difficult to predict the erosion degree of the elbow qualitatively with the existing ultrasonic detection technology, this paper combined the non-axisymmetric guided wave detection with the FrFT filtering of the LFM signal, using the PSO–LSSVM algorithm to obtain the intrinsic relationship between the time domain energy of the signals and the erosion degree, and finally realize the accurate prediction of the erosion degree of the elbow.

The LFM signal excites the PZT sensor pasted on the outer arch of one end of the elbow to generate a non-axisymmetric guided wave and collect signals through four PZT sensors arranged at the other end. The time domain energy value of the signal filtered by FrFT and the corresponding remaining wall thickness of the erosion area are used as samples to train the PSO–LSSVM model. Finally, the same test sample is used to test the model’s accuracy in predicting the erosion degree and compare it to the nonlinear regression analysis method, BP neural network, and LSSVM. The results show that the method proposed in this paper is more accurate than other methods, and it has a guiding significance for real-time monitoring of the elbow erosion.

## 2. Theoretical Background

### 2.1. The FrFT Filtering Principle of LFM Signal

As a generalized form of Fourier transform, Namias [37] redefined the concept of FrFT in a purely mathematical way from the perspective of eigenvalues and eigenfunctions, and gave a high-order differential form of FrFT in 1980. After Almeida [38] pointed out that FrFT can be understood as the rotation of the time–frequency plane, and Ozaktas [39] proposed a discrete algorithm with a calculation amount equivalent to FFT, more scholars have begun applying FrFT to the signal processing field [40,41]. The *p*-order fractional Fourier transform of a one-dimensional signal is defined as follows [42]:

(1) $$X_{p}\left(u\right)=\int_{-\infty }^{+\infty }K_{p}\left(u,t\right)x\left(t\right)dt$$

where $K_{p}(u,t)$ is the kernel function, and its expression is

(2) $$K_{p}(u,t)=\left\{\begin{array}{ll} \sqrt{1-j\cot\alpha }e^{j\pi (u^{2}\cot\alpha -2ut\csc\alpha +t^{2}\cot\alpha )}, & \alpha \neq n\pi \\ \delta (u-t), & \alpha =2n\pi \\ \delta (u+t), & \alpha =(2n\pm 1)\pi \end{array}\right.$$

where α=pπ/2 is the rotation angle, *p* is the order of the FrFT, and *δ* is the impulse function. Equation (2) shows that the FrFT kernel is essentially a set of LFM signals whose modulation frequency is cot*α*. When changing the order *p*, the rotation angle *α* changes simultaneously, and then the basis of different modulation frequencies can be obtained. An LFM signal can be represented as follows:

(3) $$x\left(t\right)=e^{j\left(2\pi f_{0}t+\pi Kt^{2}+\varphi_{0}\right)}$$

where $0\leq t\leq t_{n}$, $f_{0}$ is the initial frequency, *K* is the modulation frequency, and $\varphi_{0}$ is the initial phase of the signal. Substituting Equation (3) into Equation (1) and making cot*α* = *−K*, the *p*-order FrFT of the LFM signal can be obtained by deducing that

(4) $$X_{p}(u)=\sqrt{1+jK}e^{j\varphi_{0}-j\pi u^{2}K}\delta \left(u\csc\alpha -f_{0}\right)$$

**Figure 1:**
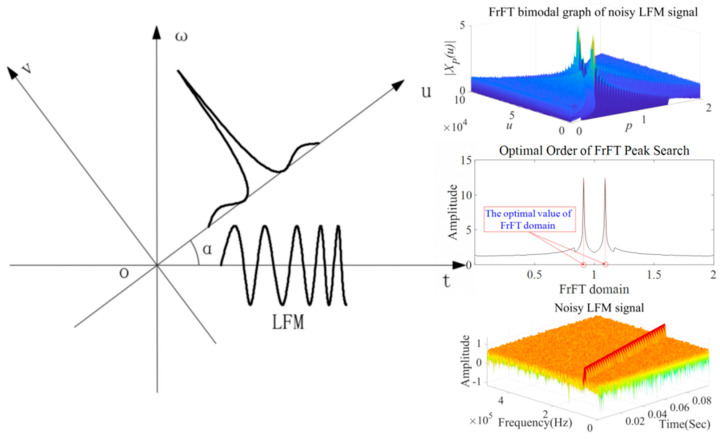
FrFT filtering principle of LFM signal.

Equation (4) shows that, within the value range of the order *p*, the continuous FrFT is performed on the noise-containing LFM signal with a certain step size. When the LFM signal’s modulation frequency is consistent with a certain set of bases, the signal’s FrFT is an impulse function that exhibits time–frequency focusing. However, noise signals do not have this property. Using this principle can realize the filtering of the LFM signal, as shown in Figure 1.

### 2.2. The Principle of LSSVM

LSSVM is an improved algorithm of SVM. Introducing an error sum of squares term into the objective function changes the inequality constraint in the quadratic programming problem to an equality constraint, which overcomes the abnormal regression caused by rough datasets and large fluctuations. It can effectively solve the problem of the standard SVM solution being too slow [43]. The regression model of LSSVM in high-dimensional space is as follows [44]:

(5) $$y(x)=\omega^{T}\varphi (x)+b$$

where $\varphi \left(x\right)$ is a non-linear mapping function that maps the input data to a high-dimensional space, making it separable; ω is the weight vector; and *b* is the bias vector. The hyperplane $\omega^{T}\varphi (x)+b=0$ can separate all samples. The optimization function of LSSVM is defined as follows:

(6) $$\min_{\omega ,\xi }J(\omega ,\xi )=\min_{\omega ,\xi }\frac{1}{2}\omega^{T}\omega +\frac{\gamma }{2}\sum_{i=1}^{N}\xi_{i}^{2}$$

where $\xi_{i}$ is the error term and γ is a regularization parameter used to balance the proportion of misclassified samples and model complexity. The constraint of this optimization function is as follows:

(7) $$y_{i}\left[(\omega^{T}x_{i})+b\right]=1-\xi_{i},i=1,2,3,\ldots ,N$$

The Lagrange function is used to solve the above optimization problem:

(8) $$L(\omega ,b,\xi_{i},\alpha_{{}_{i}})=J(\omega ,\xi )-\sum_{i=1}^{N}\alpha_{i}(y_{i}(\omega^{T}x_{i}+b)-1+\xi_{i})$$

where *α_i_* is the Lagrange multiplier. According to the KKT optimization condition, let the partial derivatives of the four parameters in ω, *b*, $\xi_{i}$, and *α_i_* in the Lagrangian function be 0, and finally obtain the LSSVM model expression:

(9) $$y\left(x\right)=sign\left[\sum_{i=1}^{N}\alpha_{i}y_{i}K\left(x,x_{i}\right)+b\right]$$

Considering that the radial basis function has the advantages of simple form and fast convergence, this paper chooses it as the kernel function of LSSVM, and its form is as follows:

(10) $$K\left(x,x_{i}\right)=\exp\left(-{\left\|x-x_{i}\right\|}^{2}/\sigma^{2}\right)$$

where σ is a kernel function parameter. The regularization parameter γ and kernel parameter σ affect the performance of LSSVM when the sample and kernel function are determined. So, obtaining the optimal global solution of these two parameters becomes the key to the problem.

### 2.3. The Principle of PSO

**Figure 2:**
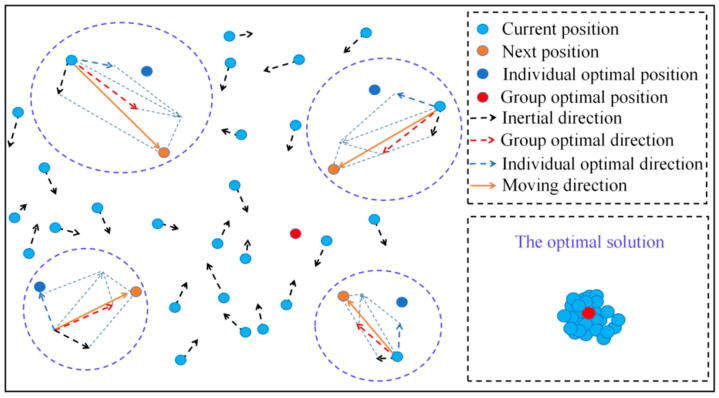
PSO algorithm.

PSO is a swarm intelligence optimization algorithm inspired by the movement of bird groups [45], which regards the group collaboration process of birds foraging as an algorithm optimization solution. The algorithm consists of a particle swarm that iteratively searches to obtain the optimal solution and find the best position, as shown in Figure 2. The principle is as follows:

Suppose there are *M* random particles in the *d*-th dimensional solution space, and the position of each particle represents a possible optimal solution. The position, velocity, and the optimal position searched so far of the *i*-th particle are set to $X_{i}=\left(x_{i1},x_{i2},\ldots ,x_{id}\right)$, $V_{i}=\left(v_{i1},v_{i2},\ldots ,v_{id}\right)$, and $P_{i}=\left(p_{g1},p_{g2},\ldots ,p_{gD}\right)$, respectively. The RMSE between the predicted value and the actual value of the remaining wall thickness of the elbow is defined as the fitness function of the particle, and the updated equations of particle velocity and position are as follows [46]:

(11) $$\left\{\begin{array}{l} v_{id}^{k+1}=\omega v_{id}^{k}+c_{1}r_{1}^{k}\left(p_{id}^{k}-x_{id}^{k}\right)+c_{2}r_{2}^{k}\left(p_{gd}^{k}-x_{gd}^{k}\right)\\ x_{id}^{k+1}=x_{id}^{k}+x_{id}^{k+1},d=1,2,\ldots ,D \end{array}\right.$$

where ω is the inertia weight and the value range is usually [0.5, 1]; $v_{id}^{k}$ and $x_{id}^{k}$ are the speed and position, respectively, of the *i*th particle in the *d*th dimension in the *k*th iteration; *c*_1_ and *c*_2_ are the learning factors; *r*_1_ and *r*_2_ are random numbers between [0, 1]; and $p_{id}^{k}$ and $p_{gd}^{k}$ are the individual optimal position of the *d*-dimensional particle *i* and the optimal global position of the entire particle swarm, respectively.

### 2.4. The Calculation Process

**Figure 3:**
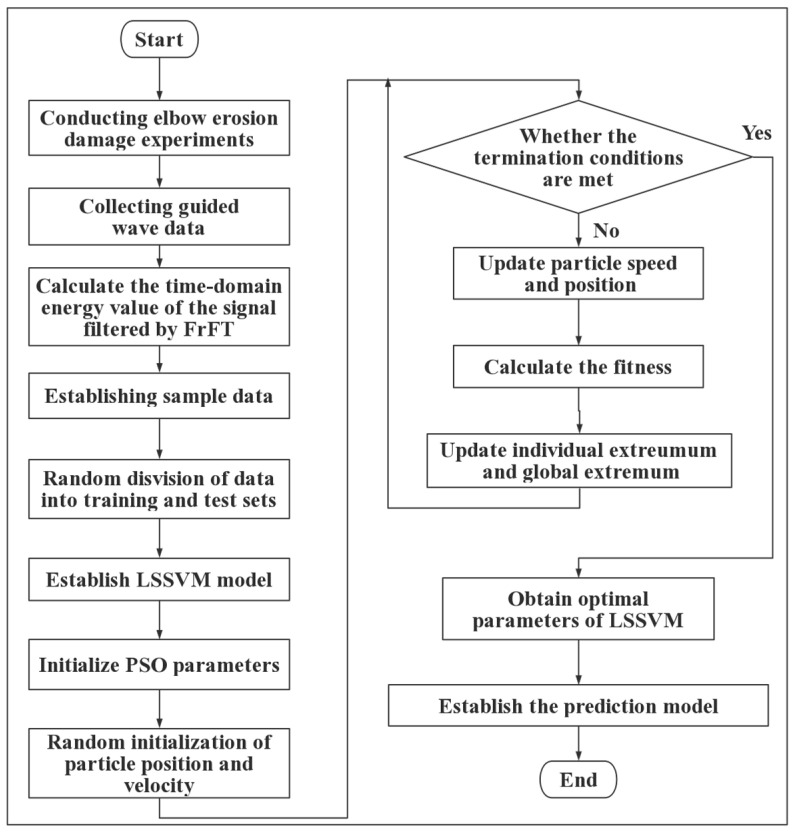
Flowchart of the prediction method process.

We proposed a prediction model for the elbow’s erosion degree based on the LFM signal’s time domain energy filtered by FrFT and the PSO-LSSVM algorithm. Figure 3 shows the algorithm flow, and the detailed steps are as follows:

1. An LFM signal is used to generate a non-axisymmetric guided wave that propagates in the 90° elbow and collects the signal through four PZT receivers arranged around the other end.
2. Perform fractional Fourier filtering on the collected signals. Use the filtered time domain energy value and the elbow’s corresponding remaining wall thickness value as sample data. The sample data are randomly divided into training and test sets.
3. Convert the regularization parameter γ and kernel parameter $\sigma^{2}$ of the LSSVM model into the two-dimensional coordinates of the particles, and initialize the parameters of the PSO algorithm.
4. When the fitness of a particle is better than the current optimal value, update its optimal fitness and record the current position; when the individual fitness of all particles is higher than the current global fitness, record the current position and update the global fitness as well as the speed and position of the particles.
5. The optimization search ends when the calculation result meets the end condition and builds the LSSVM model based on the obtained optimal global values of the regularization parameter γ and kernel parameter $\sigma^{2}$. The model is trained by taking the filtered time domain energy value of the extracted four position signals of the elbow as input and the corresponding remaining wall thickness as output.
6. The erosion degree of the elbow can be predicted according to the input sample data after the training is completed.

## 3. Experimental Setup

### 3.1. Experimental Device

**Figure 4:**
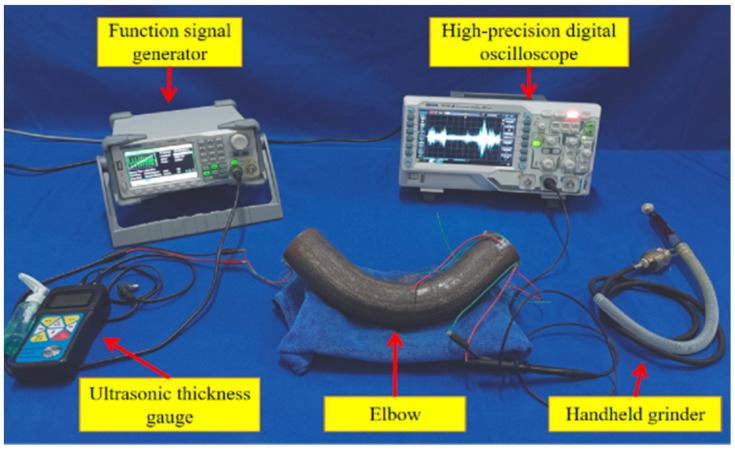
The ultrasonic testing system.

The ultrasonic testing system for elbow erosion is shown in Figure 4. It mainly comprises a function signal generator, a high-precision digital oscilloscope, a handheld grinder, and an ultrasonic thickness gauge. The detection object is a 90° metal elbow with a nominal diameter of DN = 50.8 mm and an average wall thickness of 4 mm.

### 3.2. Experimental Program

The most severe erosion area is located near the outlet end of the elbow and the overall defect morphology is symmetrical about the center plane of the elbow, showing a parabolic shape [47,48]. Therefore, we used a handheld grinder to make oval-shaped pits to simulate the actual erosion area during the experiment. The depth of grinding is roughly the same each time, and the average value of multiple measurements of the central area of the bottom of the erosion pit is measured with an ultrasonic thickness gauge [42].

**Figure 5:**
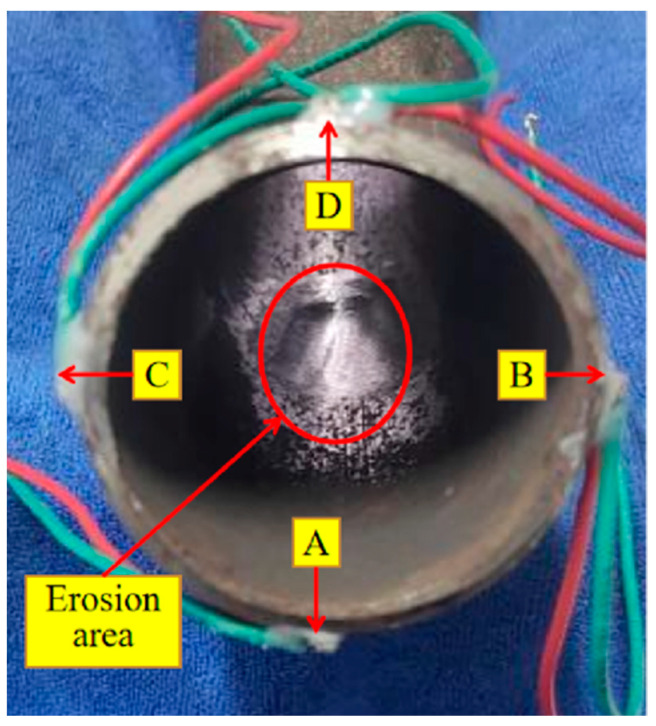
PZT paste position and the erosion area.

In practical applications, other parts are usually installed at both ends of the elbow. The collected signal energy will be small if only one end is installed with a sensor to excite and receive signals. Therefore, we chose to paste a PZT on the outer arch of one end as a signal exciter, start from the outer arch at the other end, and paste a PZT every 90° along the circumferential direction of the pipe as a signal receiver. The PZT receiver at the outer arch point is defined as PZT-A; the inner bending point is PZT-D; and the left and right ends of the connection between PZT-A and PZT-D are PZT-B and PZT-C, respectively. The artificially simulated erosion area and the PZT pasting position are shown in Figure 5. The PZT specifications and materials used in the test are the same, and the properties of the PZT material are shown in Table 1 [42].

**Figure 6:**
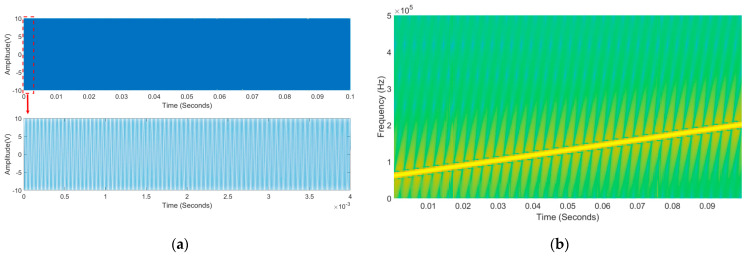
The excitation signal: (**a**) the time domain diagram; (**b**) the time–frequency diagram.

By conducting a large-scale frequency sweep test on the sample, it is determined that the excitation frequency of the LFM signal is 60–200 kHz, the signal amplitude is 10 V, the sweep time is 0.1 s, and the sampling frequency is 2 MHz. The form of the excitation signal is shown in Figure 6. During the grinding test, the sample’s artificially simulated erosion thinning process is shown in Table 2 (C0 is the unpolished state).

### 3.3. Time Domain and Energy Analysis of Signals

**Figure 7:**
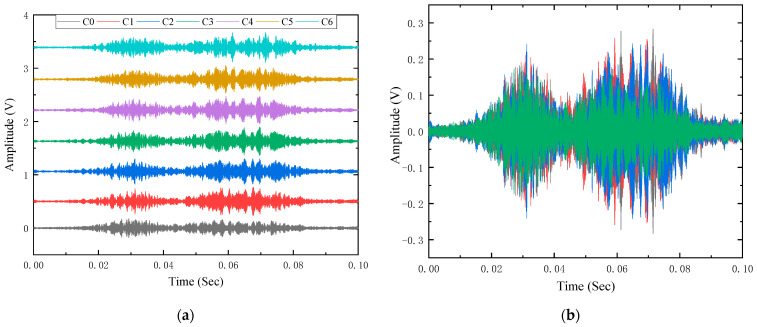
The time domain diagram of the received signal by PZT-A: (**a**) time domain signals of each erosion degree; (**b**) time domain signal synthesis diagram of multiple erosion degrees.

Taking the signal received by PZT-A as an example, Figure 7a is the offset diagram of the received signal under different erosion degrees, and Figure 7b is the time domain signal synthesis diagram of erosion degrees C0, C2, C4, and C6. It can be found that there is no significant difference in the time domain signal waveform and amplitude under different erosion degrees. It is difficult to directly obtain the relationship between the erosion degree and the received signals in the time domain, and further analysis is required.

**Figure 8:**
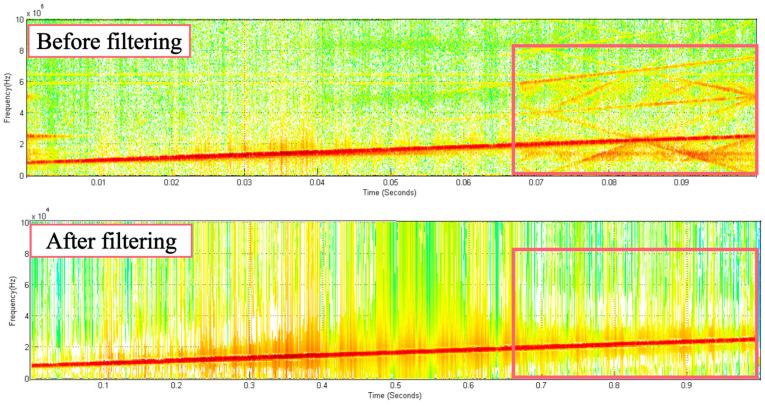
Time–frequency diagram of STFT before and after filtering.

Taking the signal collected by PZT-A when the erosion degree is C0, the STFT is performed on the signals before and after FrFT filtering, and the time–frequency diagram is obtained, as shown in Figure 8. The noise signals in the original signal are effectively filtered out, and the LFM signal is well preserved. This shows that FrFT can effectively filter out the noise in the acquisition signal, thereby reducing the error caused by the noise.

**Figure 9:**
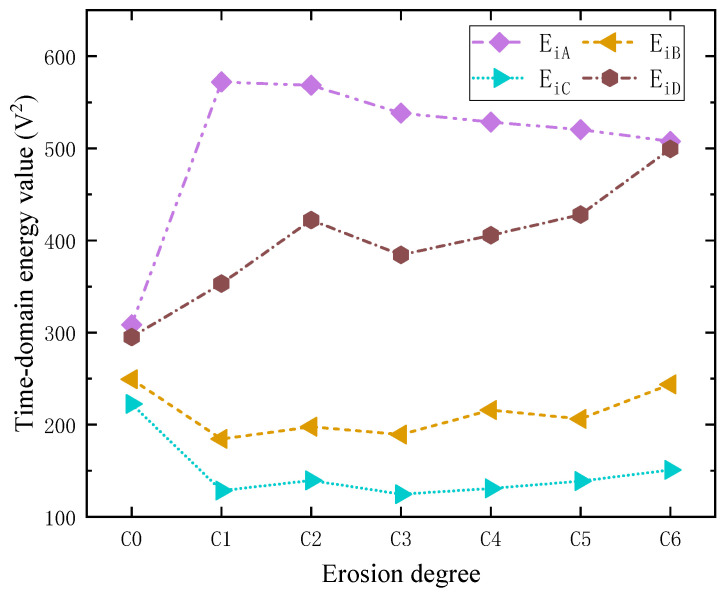
The time domain energy values after filtering for each received position signal under different erosion degrees.

Calculate the time domain energy value of the signals after FrFT filtering and represent them with *E_iA_*, *E_iB_*, *E_iC_*, and *E_iD_* (*i* = 0~6 represents seven erosion degrees. A, B, C, and D represent the four receiving positions). Take the average value of the time domain energy at the same erosion degree and position, and construct a graph as shown in Figure 9.

As the erosion degree increases, the reflected guided wave at the erosion area gradually increases and the time domain energy of the signal at point A of the outer arch generally shows a downward trend, but there are also cases of abnormal energy (such as when the erosion degree is C1). The erosion area is on the shortest path between the guided wave exciting point and the outer arch PZT-A. In erosion degree C0, the metal oxide layer on the inner surface of the erosion area reduces the time domain energy of the signal received by PZT-A. In erosion degree C1, the metal oxide layer in this area is removed. Therefore, the time domain energy of the signal collected by the outer arch back PZT-A is higher than that of erosion degree C0.

After the non-axisymmetric guided wave is excited, the guided wave will form a wavefront in the elbow and propagate to the surroundings. The sources of the guided wave signals received by PZT-B and PZT-C are complex, and the erosion area is not on the minimum path between the two PZT sensors and the excitation point. Therefore, there is no apparent correspondence between the energy values of PZT-B and PZT-C acquisition signals and the surface morphology and depth of the erosion area. With increasing erosion, the guided wave is refracted and reflected in the erosion area, and its propagation path changes. For the elbow measured in this test, the specific performance is that the time domain energy of the signal received by PZT-D of the inner bend gradually increases.

The above results show that, for the elbow in this test, there is a correspondence between the time domain energy of the signals received by PZT-A on the back of the outer arch and PZT-D on the inner bend and the erosion degree. However, it is difficult to accurately predict the erosion degrees of the elbow from the energy point of view alone.

## 4. Establishment of the Prediction Model for Erosion Degree of the Elbow

### 4.1. Extract Sample Set

Extracting more signal data for each erosion degree to train the prediction model can effectively reduce the variability in signal energy caused by experimental measurement errors. In this experiment, the four PZT receivers collected 20 sample datasets at each erosion degree (10 sets at erosion degree C6). In order to better verify the regression ability of the model, the remaining thicknesses were measured multiple times under each erosion degree and corresponded to the time domain energy value of the signals collected from the four PZTs after FrFT filtering; partial sample data are shown in Table 3. Under the same erosion degree, the difference between the time domain energy of the signal collected by the same PZT sensor is much smaller than the difference between different erosion degrees. Expanding the training sample by measuring the ultrasonic signal multiple times can reduce the error in the signal acquisition process as much as possible.

### 4.2. PSO–LSSVM Model

The PSO algorithm is used to find the optimal value of the regularization parameter γ and kernel parameter $\sigma^{2}$ iteratively. The initialization settings of relevant PSO parameters are shown in Table 4, and using random functions generate the initial position and velocity of particles. After 30 iterations, the optimal global values of these two parameters are obtained and the PSO–LSSVM model is established.

### 4.3. Predict the Erosion Degree of Elbow

**Figure 10:**
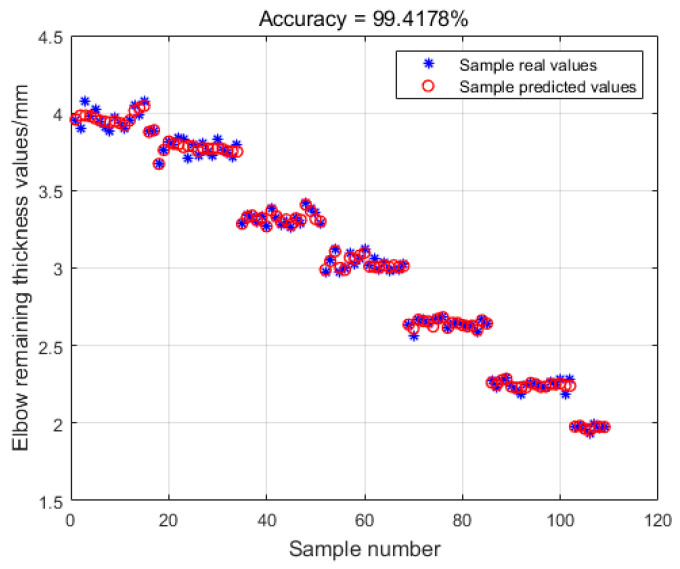
Comparison of the predicted values of the training set sample and the real values.

In order to test the regression fitting effect, the training set data are input into the model, and the comparison between the real value and the predicted value of the training sample is shown in Figure 10.

**Figure 11:**
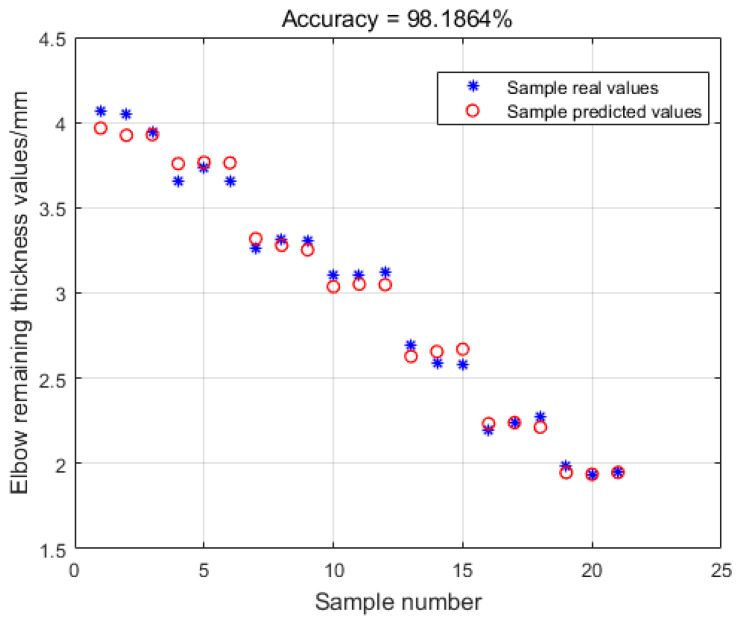
Comparison of the predicted values of the test set sample and the actual values.

Figure 10 shows that the model has excellent learning and regression-fitting abilities on the training set, with an accuracy rate of 99.4178%. In order to test the predictive ability of the model, 21 sets of test set data were input for prediction, and the results are shown in Figure 11. The model predicts with 98.1864% accuracy on the test set, indicating that the model can accurately predict the remaining thickness value of the elbow.

## 5. Comparison and Analysis

### 5.1. Other Methods

In order to further verify the effectiveness and accuracy of the method proposed in this paper in predicting the erosion degree of the elbow, we use the same experimental data as input and predict the remaining thickness using the nonlinear regression analysis method, LSSVM algorithm, and BP neural network.

When using the nonlinear regression analysis method, the independent variables are the time domain energy values of the signals after FrFT filtering received by the four PZT receivers, and the regression model formula is defined as follows:

(12) $$t=CE_{iA}{}^{a_{1}}E_{iB}{}^{a_{2}}E_{iC}{}^{a_{3}}E_{iD}{}^{a_{4}}$$

where *C*, *a*_1_, *a*_2_, *a*_3_, and *a*_4_ are the regression coefficients and *t* is the predicted remaining thickness value. Substitute the training set data into Equation (12) to establish the nonlinear relationship between 109 groups of the independent variable and the remaining thickness. This set of multiple regression equations is solved to obtain the values of each regression coefficient and establish the nonlinear regression empirical equation for the prediction of the remaining thickness of the elbow:

(13) $$t=E_{iA}{}^{1.1206}E_{iB}{}^{-1.1338}E_{iC}{}^{-1.2588}E_{iD}{}^{1.5476}$$

In the LSSVM algorithm, the kernel function is the radial basis function shown in Equation (10). The value ranges of the regularization parameter γ and the kernel function parameter $\sigma^{2}$ are set to be [0, 300] and [0, 200], respectively. The grid method is used to fold the value range of the two parameters ten times to obtain their optimal value and input it into the LSSVM. The LSSVM model is trained using the training set data.

When building the model based on the BP neural network, the input is the feature matrix of the training set samples and the output is the predicted value of the remaining thickness. The model uses a three-layer neural network, and its related parameter settings are shown in Table 5.

### 5.2. Results and Discussion

**Figure 12:**
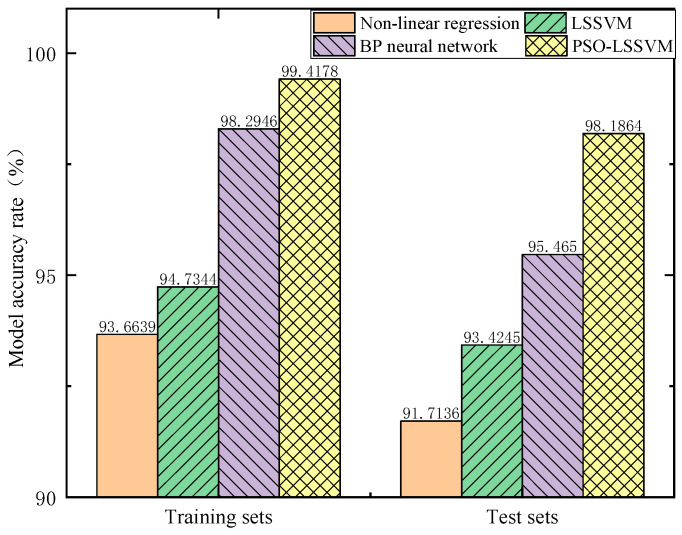
Comparison of model prediction accuracy.

Input the sample data into the above models to obtain the prediction accuracy of each model and compare it to PSO–LSSVM, as shown in Figure 12. The results show that the prediction accuracy of the training set samples by the four methods is higher than that of the test set samples under the same circumstances, indicating that the above models all have good regression fitting performance. PSO–LSSVM has the highest prediction accuracy for training and test samples, at 99.4178% and 98.1864%, respectively.

In order to verify the applicability of the method proposed in this paper, we used the same experimental method to test the two other elbows (the nominal diameters of elbow 2 and elbow 3 are 50.8 mm and 108 mm, respectively, and the average thicknesses are 4 mm and 8 mm, respectively) and analyzed the extracted data.

**Figure 13:**
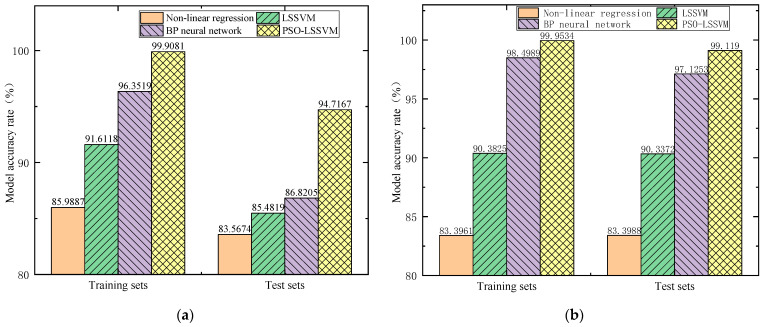
Comparison of the prediction accuracy of each method model for two elbows: (**a**) elbow 2 and (**b**) elbow 3.

Figure 13 shows the accuracy of the above four prediction models for the remaining thickness of the two elbows. Both the nonlinear regression model and the LSSVM model have lower accuracy in predicting the remaining thickness of the elbows. The prediction accuracy of the BP neural network model on the test set data of Elbow 2 and Elbow 3 is 86.8205% and 97.1253%, respectively, which shows that its predictive ability is not stable enough. The PSO–LSSVM model has the highest and most stable prediction accuracy for the remaining thickness of these two elbows, reaching 94.7167% and 99.119%, respectively.

In order to test the accuracy of the prediction of the new erosion degree, we conducted a test on a DN219 elbow. We extracted 20 sets of data under eight erosion degrees for analysis. The remaining wall thickness values are shown in Table 6. The sample set under each erosion degree is randomly divided into the training set and test set according to the ratio of 4:1. Three different training sets and test set sample features are used to verify the method proposed in this paper, as shown in Table 7.

**Figure 14:**
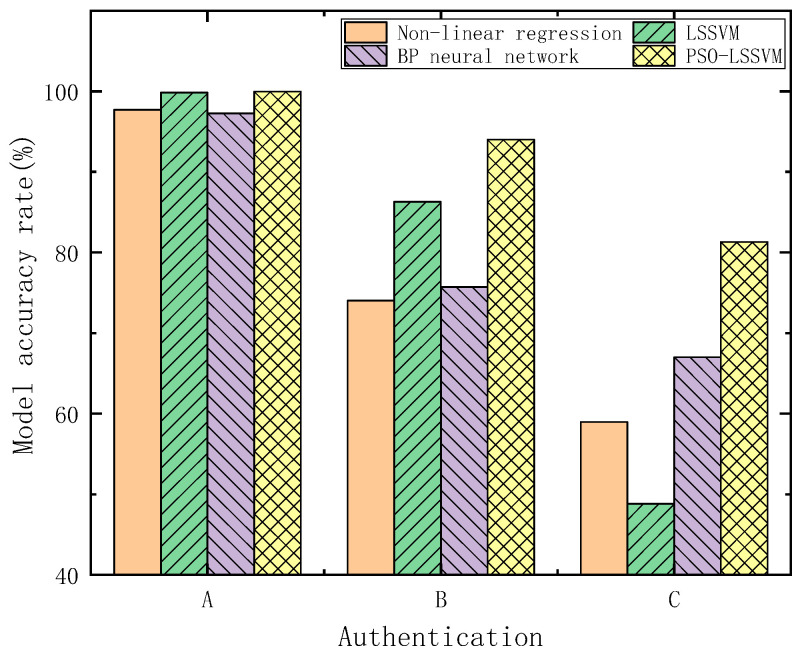
The accuracy of each model for the three verification methods.

The accuracy rates of each model are shown in Figure 14. It can be seen that, under these three verification methods, the accuracy rates of PSO-LSSVM are 99.9593%, 94.0039%, and 81.2976%, respectively, which are still higher than those of the other methods.

Measurement errors during experimentation, ambient noise, and sample parameters used to train the model can affect predictions. The measurement error mainly comes from the influence of the PZT sensor pasting process. Therefore, during the test, it should be ensured that the glue layer between the PZT sensor and the elbow is as thin as possible under the premise of insulation, and it should be ensured that the sensor has been firmly pasted on the elbow before the test. FrFT can filter the error caused by environmental noise in the method proposed, so it does not need to be considered. Increasing the erosion state and the number of samples contained in the model training samples can improve the model’s accuracy.

## 6. Conclusions and Future Work

1. In this paper, the LFM signal is used to excite the non-axisymmetric guided wave to detect the erosion degree of the elbow, and the collected signals are filtered by FrFT. The results show that, with the increase in the erosion degree, the time domain energy of the position signal of the outer arch gradually decreases, the time domain energy of the position signal of the inner bend gradually increases, and the time domain energy variation of the symmetrical position signals on both sides is not apparent.
2. A prediction method of the erosion degree of the elbow based on FrFT and PSO-LSSVM model is proposed. The accuracy on the test set samples of the four elbows reached 98.1864%, 94.7167%, 99.119%, and 99.9593% respectively, and the accuracy of predicting the two new erosion degrees is 94.0039% and 81.2976%, respectively, which is better than the nonlinear regression analysis method, LSSVM, and BP neural network algorithm.
3. The method proposed in this paper has successfully realized the prediction of the erosion degree of the elbow under laboratory conditions. In the follow-up work, we will optimize the detection method proposed in this paper according to the actual engineering situation, study the influence of the characteristic parameters of the signal on the prediction accuracy of the elbow erosion degree, and finally realize the real-time monitoring of the pipeline elbow in practical application.

## Figures and Tables

**Table 1:** The properties of PZT.

Property	Value	Property	Value
Density	7.5 g/cm^3^	Curie temperature	340 °C
Relative dielectric constant	1720	Mechanical quality factor	600
Electromechanical coupling coefficient	0.6	Frequency constants	1990 Hz·m
Piezoelectric coefficient	450 × 10^12^ C/N	Dielectric loss	0.02

**Table 2:** The remaining thickness values under each erosion degree.

Erosion Degree	Remaining Thickness/mm
C0	3.98
C1	3.75
C2	3.34
C3	3.05
C4	2.63
C5	2.24
C6	1.97

**Table 3:** Partial sample set data table.

Sample Number	*E_iA_*/V^2^	*E_iB_*/V^2^	*E_iC_*/V^2^	*E_iD_*/V^2^	Remaining Thickness Values/mm
C0-1	315.8	250.3	286.6	220.1	3.95
C0-2	317.2	250.7	288.1	220.5	3.90
C0-3	317.5	250.6	287.9	220.6	4.08
…					
C3-1	580.3	190.3	383.3	124.5	2.98
C3-2	579.9	190.5	383.9	124.5	3.06
C3-3	555.0	190.6	383.9	124.4	3.12
…					
C6-8	520.0	243.6	500.0	151.1	1.97
C6-9	515.3	243.8	500.6	151.2	1.98
C6-10	515.8	243.7	499.9	151.2	1.96

**Table 4:** PSO parameter settings.

Sample Number	Remaining Thickness Values/mm
Iterations	30
Particle number	200
Learning factor *c*_1_, *c*_2_	0.5, 0.5
Inertia weight ω	0.95, 0.4
The value range of γ	0.01~300
The value range of $\sigma^{2}$	0.01~200

**Table 5:** BP neural network parameter settings.

Property	Value
Number of hidden layers	1
Number of neurons	10
Network convergence accuracy	0.001
Maximum number of iterations	100
Learning algorithm	L-M optimization algorithm
Number of hidden layers	1

**Table 6:** The remaining thickness values under each erosion degree of DN219 elbow.

Erosion Degree	Remaining Thickness/mm
C0	9.67
C1	8.53
C2	7.72
C3	6.90
C4	6.03
C5	5.07
C6	4.25
C7	2.91

**Table 7:** Verification methods for different training sets and test sets.

Verification Method	Training Set	Test Set
A	All erosion degrees	All erosion degrees
B	All erosion degrees except C3	C3
C	All erosion degrees except C7	C7

## Data Availability

Not applicable.
